# Retraction Note: Preservation of dendritic spine morphology and postsynaptic signaling markers after treatment with solid lipid curcumin particles in the 5xFAD mouse model of Alzheimer’s amyloidosis

**DOI:** 10.1186/s13195-023-01313-7

**Published:** 2023-09-29

**Authors:** Panchanan Maiti, Zackary Bowers, Ali Bourcier-Schultz, Jarod Morse, Gary L. Dunbar

**Affiliations:** 1 Field Neurosciences Institute Laboratory for Restorative Neurology, Central Michigan University, Mt. Pleasant, MI 48859 USA; 2 Program in Neuroscience, Central Michigan University, Mt. Pleasant, MI 48859 USA; 3 Department of Psychology, Central Michigan University, Mt. Pleasant, MI 48859 USA; 4 Field Neurosciences Institute, Ascension St. Mary’s Hospital, Saginaw, MI 48604 USA; 5 College of Health and Human Services, Saginaw Valley State University, Saginaw, MI 48710 USA

**Retraction Note: Alz Res Ther 13, 37 (2021)**

**https://doi.org/10.1186/s13195-021-00769-9**

The Editors-in-Chief have retracted this article at the authors' request. After publication, concerns were raised regarding a number of cases of image overlap within the Figures, which the authors addressed by a Correction [1]. However, subsequent concerns have been raised about further image similarities within the article, as well as with previous studies. Specifically:

- Fig. 3b 5xFAD + Veh CAI 12-month-old group image appears highly similar to an image representing the same group in [2].
- In Fig. 3B, 5xFAD + SCLP Cortex and Entorhinal cortex images appear to originate from the same sample.
- In Fig. 3E, the beta-tubulin blots presented for the cortex 6- and 12-month-old groups appear highly similar; the beta-tubulin blot image for hippocampus in the 6-month-old group appears highly similar to that representing beta-tubulin in the 12-month-old group in Figs. 5F and 7A.
- The updated Fig. 4G 5xFAD Primary 6-month-old group image appears to overlap with Fig. 4I 5xFAD Secondary 12-month-old group.
- The top beta-tubulin blot in Fig. 6F appears highly similar to the beta-tubulin (hippocampus) blot in Fig. 7A.
- The blots in Fig. S2I appear highly similar to those in Fig. 3D of [3, now retracted].
- Fig. S5A appears highly similar to the Merged 5xFAR + SLCP (2d) image in Fig. 1B of [3, now retracted].

Despite internal data validation, the authors no longer have sufficient confidence in the findings of this study.

All authors agree to this retraction.
